# Effects of Long-Term Dietary Inclusion of Citrus Pomace on Growth Performance, Intestinal Morphology, Digestive Enzyme Activity, Antioxidant Status, and Colonic Microbiota in Tibetan Pigs

**DOI:** 10.3390/ani15162348

**Published:** 2025-08-11

**Authors:** Xiaobo Guo, Haopeng Zhong, Jianjun Li, Xiaocui Lin, Yan Hu, Guosheng Zhang, Jun Chen, Jinming You

**Affiliations:** 1 Jiangxi Province Key Laboratory of Animal Nutrition and Feed, Jiangxi Province Key Innovation Center of Integration in Production and Education for High-Quality and Safe Livestock and Poultry, Jiangxi Agricultural University, Nanchang 330045, China; 2 Ganzhou Animal Husbandry and Fisheries Research Institute, Ganzhou 341000, China; 3 Gannan Academy of Sciences, Ganzhou 341000, China; 4 Jiangxi Agricultural Technology Extension Center, Nanchang 330045, China

**Keywords:** antioxidant status, citrus pomace, colonic microbiota, digestive enzyme activity, growth performance, intestinal morphology, Tibetan pigs

## Abstract

This study aimed to evaluate the impacts of dietary inclusion of citrus pomace on growth performance, intestinal morphology, digestive enzyme activity, antioxidant status, and colonic microbiota in Tibetan pigs in a 90-day feeding trial. Eighty Tibetan pigs (75-day-age, 16.62 ± 1.50 kg) were divided into four dietary treatment groups, each containing four replicates with five pigs per replicate. The experimental diets comprised a control diet alongside three diets incorporating 5%, 10%, or 15% citrus pomace, respectively. The results showed that the feed conversion ratio of pigs was lower in the 5% citrus pomace group compared to the other three treatment groups. Compared to the control group, dietary inclusion of 5% citrus pomace increased villus height in the duodenum and jejunum of pigs. Additionally, it elevated β-amylase activity in the duodenum while increasing sucrase activity in the jejunum. Furthermore, dietary inclusion of 5% citrus pomace enhanced lipase activity in the duodenum, jejunum, and ileum of pigs compared to the control group. It also significantly increased catalase activity in the serum of pigs. However, dietary inclusion of 5% citrus pomace decreased the abundances of *Lactobacillaceae* and *Lactobacillus* while increasing the abundances of *Streptococcaceae*, *Turicibacteraceae*, *Streptococcus*, and *Turicibacter* in the colonic digesta of pigs. Collectively, long-term dietary inclusion with 5% citrus pomace reduced the feed conversion ratio, improved intestinal morphology, enhanced digestive enzyme activity in the small intestine, and elevated serum antioxidant status, while exerting complex effects on colonic microbiota in Tibetan pigs. Future research should prioritize in vitro fermentation to develop fermented citrus pomace as a feed ingredient for pigs.

## Simple Summary

The global demand for grain-based feed in the livestock sector has risen significantly in recent years. The utilization of food processing by-products as alternative feed ingredients presents a viable strategy to mitigate this issue. Citrus pomace, a residual by-product derived from juice and citrus product manufacturing, exhibits considerable promise as a dietary component for swine due to its high nutritional value and bioactive constituents. Findings from this study suggest that a 5% inclusion of citrus pomace in the diet of Tibetan pigs is sustainable over extended periods.

## 1. Introduction

The global demand for grain production in the livestock industry has increased in recent years. Utilizing food by-products as feed ingredients has emerged as a potential solution to address this challenge [1]. Citrus pomace, an agricultural by-product from juice or citrus-based product processing, demonstrates significant potential as a pig feed ingredient due to its rich traditional nutrient content and bioactive compounds [2]. Citrus fruit processing generates substantial by-products, including peels, pulp, membranes, and seeds [3]. While these by-products pose economic and environmental challenges, they can also be utilized as valuable dietary components for ruminants and rabbits [4,5]. Researchers have prioritized by-product utilization to effectively reduce environmental impacts, alleviate competition for food and feed resources, and optimize land use [1].

According to the World Citrus Organization, China accounts for approximately 28% of global citrus production, establishing itself as a leader in both citrus orchard area and output worldwide [6]. Citrus fruits are rich in traditional nutrients, including carbohydrates, proteins, lipids, vitamins, and minerals, as well as bioactive compounds such as flavonoids, essential oils, carotenoids, limonoids, and coumarins [7]. Citrus pulp has demonstrated antioxidant and antimicrobial properties [8,9]. In poultry production, dietary inclusion of dried sweet orange (*Citrus sinensis*) pulp enhanced growth performance (feed intake and body weight gain) while reducing hepatic and abdominal fat deposition in broiler chickens [10]. Additionally, dietary supplementation with 2% Citrus junos probiotics enhanced immune function and reduced thiobarbituric acid reactive substances in broiler breast meat [11]. In growing rabbits, citrus pomace consumption showed no adverse effects on growth performance but instead improved immunity and hepatic antioxidant status [12]. Furthermore, the Tibetan pig, a native Chinese breed with herbivorous tendencies, has more diverse intestinal microbiota, characterized by a high abundance of fiber-degrading bacteria in the large intestine. These functional microorganisms are capable of decomposing cellulosic and other complex substances present in roughage, thereby facilitating the host’s nutrient digestion and absorption [13]. Growing pigs can adapt their gastrointestinal tract to efficiently utilize citrus pulp as a source of highly fermentable carbohydrates [14,15]. However, limited information is available regarding the impact of long-term dietary citrus pulp inclusion on the growth performance, antioxidant status, and intestinal health of Tibetan pigs.

Therefore, this study was conducted to investigate the effects of dietary inclusion of citrus pomace on growth performance, intestinal morphology, digestive enzyme activity, antioxidant status, and colonic microbiota of Tibetan pigs in a 90-day feeding trial.

## 2. Materials and Methods

### 2.1. Experimental Design

The animal protocols were approved by the Animal Care and Use Committee of Jiangxi Agricultural University (JAXULL-0132). Eighty 75-day-old Tibetan pigs, with an average body weight of 16.62 ± 1.50 kg, were assigned to four dietary treatments, each with four replicate pens, and each pen housing five pigs. The study included four experimental diets: a control diet and three diets supplemented with 5%, 10%, or 15% citrus pomace. The citrus pomace used in this study was sourced from the by-product of Newhall Navel oranges produced in Jiangxi Province, the primary production area for Newhall Navel oranges in China. The ingredient composition and nutrient levels of the experimental diets are presented in Table 1. The feeding trial lasted 90 days, during which pigs had ad libitum access to feed and fresh water.

### 2.2. Data and Sample Collection

#### 2.2.1. Growth Performance

At the beginning and end of the feeding trial, pigs were weighed to determine the average daily gain (ADG). Feed intake per pen was recorded to calculate the average daily feed intake (ADFI). The feed conversion ratio (FCR) was determined accordingly.

#### 2.2.2. Sample Collection

At the end of the trial, one pig with a body weight similar to the average body weight in each pen was selected for sampling in the control group and the 5% citrus pomace group (the 5% citrus pomace group was selected based on growth performance). Firstly, following a 12 h fasting period, one pig per pen, as described above, was sampled for blood collection via the anterior vena cava. The blood samples were centrifuged at 4 °C and 3000 rpm for 15 min to harvest serum, which was promptly frozen in liquid nitrogen and stored at –80 °C until subsequent analysis of antioxidant parameters. Subsequently, the pigs were euthanized via intravenous injection of pentobarbital sodium. The intestinal tract was promptly excised and segmented into the duodenum, jejunum, and ileum. Samples of 2 g intestinal contents from each segment were collected and stored at –80 °C for subsequent analysis of digestive enzyme activity. Segments approximately 2 cm from the mid-portions of the duodenum, jejunum, and ileum were sampled and fixed in 4% paraformaldehyde for morphological analysis. Finally, the colonic digesta was collected and preserved in liquid nitrogen for subsequent microbiota analysis.

### 2.3. Laboratory Analysis

#### 2.3.1. Intestinal Morphology

The intestinal morphology was determined as described in our previous study [16]. Briefly, the intestinal segments were fixed, dehydrated, embedded in paraffin, and sectioned. The sections were then deparaffinized, rehydrated, and stained with hematoxylin and eosin (H&E). Digital images of the H&E-stained sections were acquired using an EVOS microscope (Advanced Microscopy Group, Bothell, WA, USA). The villus height and crypt depth were measured using Image-Pro Plus 6.0 software, and the villus height-to-crypt depth ratio was subsequently calculated using the formula: villus height divided by crypt depth.

#### 2.3.2. Digestive Enzyme Activity in the Small Intestine

Approximately 1 g of frozen duodenum, jejunum, or ileum digesta samples was weighed and homogenized with nine volumes (*w*/*v*) of precooled physiological saline. The mixture was centrifuged at 4000× *g* for 10 min at 4 °C. The supernatant protein concentration was determined using a protein quantification kit (Nanjing Jiancheng Bioengineering Institute, Nanjing, China). Subsequently, the activities of α-amylase, β-amylase, sucrase, lipase, and neutral protease in the supernatant were measured using commercial kits (Nanjing Jiancheng Bioengineering Institute, Nanjing, China) following the manufacturer’s instructions.

#### 2.3.3. Serum Antioxidant Parameters

The serum antioxidant parameters, including glutathione peroxidase (GSH-Px) activity, superoxide dismutase (SOD) activity, catalase (CAT) activity, and malondialdehyde (MDA) content, were measured using commercial assay kits (Sino Best Biological Technology, Shanghai, China) following the manufacturer’s specifications.

#### 2.3.4. Colonic Microbiota Analysis

Genomic DNA was extracted from colonic digesta using the OMEGA Soil DNA Kit (Omega Bio-Tek, Norcross, GA, USA). The V3–V4 region of bacterial 16S rRNA genes was amplified via PCR with primers 338F (5′-ACTCCTACGGGAGGCAGCA-3′) and 806R (5′-GGACTACHVGGGTWTCTAAT-3′). Sample-specific 7 bp barcodes were integrated into the primers for multiplex sequencing. The amplicons were pooled in equimolar amounts, and paired-end sequencing was performed on the Illumina NovaSeq platform using the NovaSeq 6000 SP Reagent Kit (500 cycles). Data processing and bioinformatics analyses were carried out by Shanghai Personal Biotechnology Co., Ltd. (Shanghai, China).

### 2.4. Statistical Analysis

Data were analyzed using SPSS 26.0 software (SPSS, Chicago, IL, USA). For the growth performance data, each pen was considered the experimental unit, and one-way analysis of variance was performed, followed by Duncan’s multiple range test for multiple comparisons among treatments. All other variables, excluding growth performance, were analyzed using a *t*-test, with each sampled pig per pen serving as the experimental unit. A significance level of *p* < 0.05 was considered statistically significant.

## 3. Results

### 3.1. Growth Performance

As shown in Table 2, no statistical differences were observed in the final body weight and average daily gain of pigs among the four dietary treatment groups (*p* > 0.05). However, compared with the control group without citrus pomace inclusion, pigs in the 10% citrus pomace group exhibited increased average daily feed intake, while those in the 5% citrus pomace group showed a reduced feed conversion ratio (*p* < 0.05). Therefore, pigs from the control group and the 5% citrus pomace inclusion group were selected for subsequent analysis of intestinal morphology, digestive enzyme activity in the small intestine, serum antioxidant status, and colonic microbiota.

### 3.2. Intestinal Morphology

**Figure 1:**
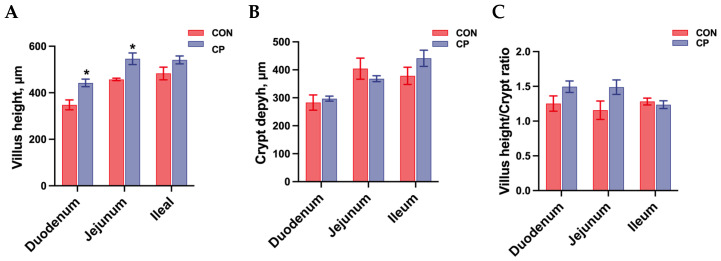
Effects of dietary inclusion with 5% citrus pomace on intestinal morphology of Tibetan pigs (n = 4, mean ± SEM): (**A**). Villus height. (**B**). Crypt depth. (**C**). Villus height/crypt depth ratio. * *p* < 0.05. Abbreviations: CON, control group; CP, 5% citrus pomace group.

The effects of dietary inclusion with 5% citrus pomace on intestinal morphology in Tibetan pigs are shown in Figure 1. No statistical differences were found in crypt depth and the villus height-to-crypt depth ratio of the duodenum, jejunum, and ileum in Tibetan pigs (*p* > 0.05). However, compared to the control group without citrus pomace inclusion, dietary inclusion of 5% citrus pomace increased villus height in the duodenum and jejunum of Tibetan pigs (*p* < 0.05).

### 3.3. Digestive Enzyme Activity in Small Intestine

**Figure 2:**
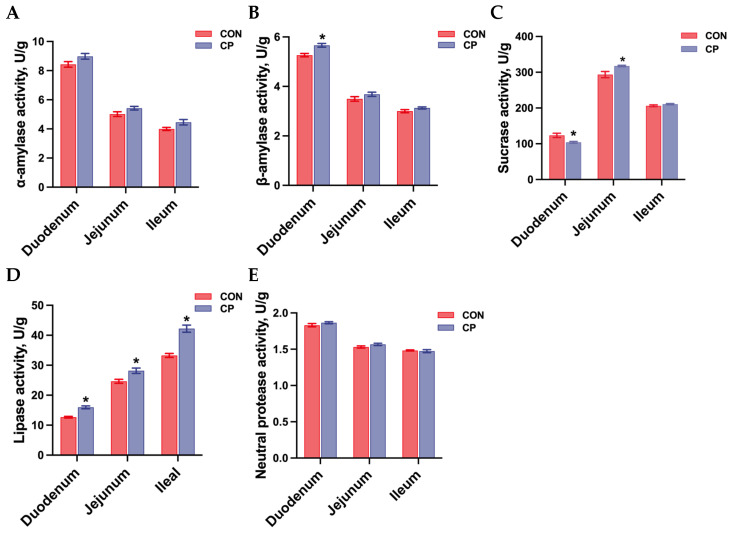
Effects of dietary inclusion with 5% citrus pomace on digestive enzyme activity in the small intestine of Tibetan pigs (n = 4, mean ± SEM): (**A**). α-amylase activity. (**B**). β-amylase activity. (**C**). Sucrase activity. (**D**). Lipase activity. (**E**). Neutral protease activity. * *p* < 0.05. Abbreviations: CON, control group; CP, 5% citrus pomace group.

As illustrated in Figure 2, no statistical differences were observed in α-amylase activity, and neutral protease activity in the small intestine of Tibetan pigs between treatment groups (*p* > 0.05). However, compared with the control group, dietary inclusion of 5% citrus pomace increased β-amylase activity and decreased sucrase activity in the duodenum, while increasing sucrase activity in the jejunum of Tibetan pigs (*p* < 0.05). Moreover, dietary inclusion of 5% citrus pomace elevated lipase activity in the duodenum, jejunum, and ileum of Tibetan pigs compared to the control group (*p* < 0.05).

### 3.4. Serum Antioxidant Status

**Figure 3:**
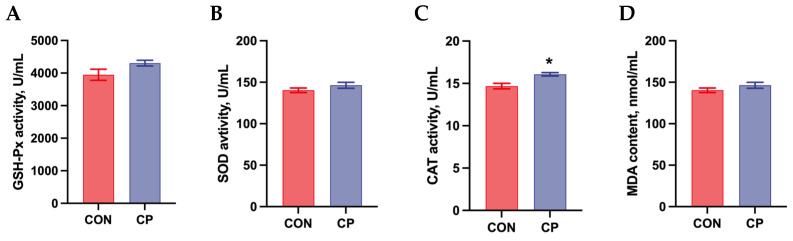
Effects of dietary inclusion with 5% citrus pomace on antioxidant status in the serum of Tibetan pigs (n = 4, mean ± SEM): (**A**). Glutathione peroxidase (GSH-Px) activity. (**B**). Superoxide dismutase (SOD) activity. (**C**). Catalase (CAT) activity. (**D**). Malondialdehyde (MDA) content. * *p* < 0.05. Abbreviations: CON, control group; CP, 5% citrus pomace group.

The effects of dietary inclusion of 5% citrus pomace on the antioxidant status in the serum of Tibetan pigs are shown in Figure 3. No significant differences were observed in GSH-Px activity, SOD activity, and MDA content in the serum of Tibetan pigs (*p* > 0.05). However, compared to the control group, dietary inclusion of 5% citrus pomace significantly increased CAT activity in the serum of Tibetan pigs (*p* < 0.05).

### 3.5. Diversity of Colonic Microbiota

**Figure 4:**
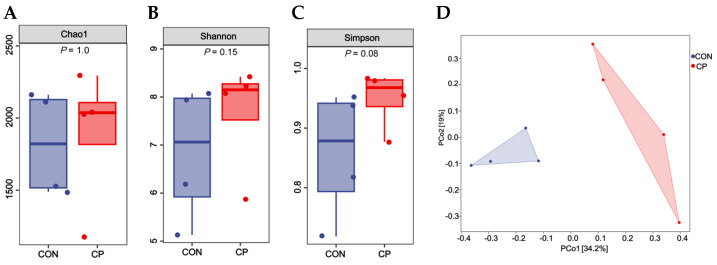
Effects of dietary inclusion with 5% citrus pomace on the diversity of colonic microbiota in Tibetan pigs (n = 4): (**A**). Chao1 index. (**B**). Shannon index. (**C**). Simpson index. (**D**). β-diversity. Abbreviations: CON, control group; CP, 5% citrus pomace group.

As shown in Figure 4, there were no statistical differences in the Chao1 index, Shannon index, and Simpson index between treatment groups (*p* > 0.05). However, a significant difference was found in β-diversity between treatment groups (*p* < 0.05).

### 3.6. Composition of Colonic Microbiota

**Figure 5:**
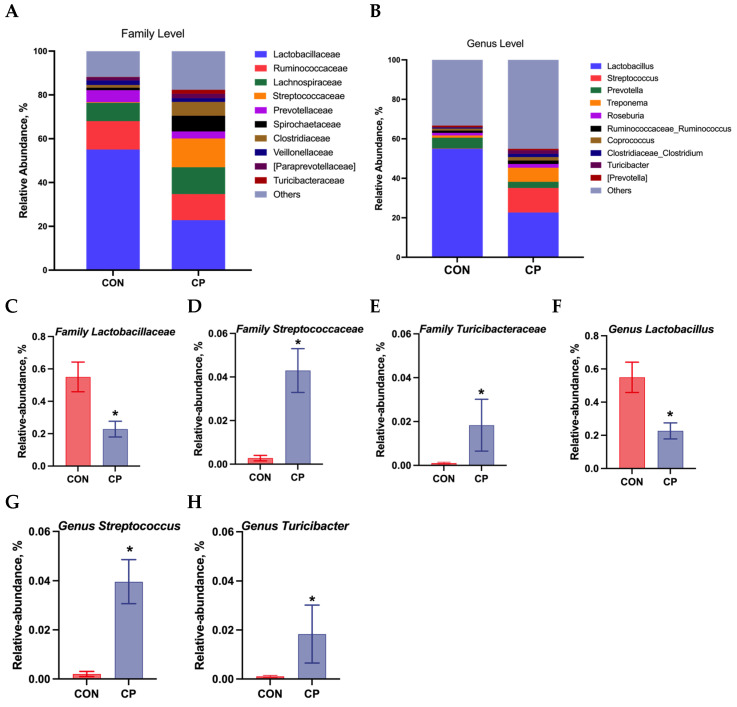
Colonic microbiota composition in Tibetan pigs fed the two experimental diets: (**A**,**B**). Relative abundance at family and genus levels (n = 4). (**C**–**H**). Relative abundance of *Lactobacillaceae*, *Streptococcaceae*, *Turicibacteraceae*, *Lactobacillus*, *Streptococcus*, and *Turicibacter* (n = 4, mean ± SEM). * *p* < 0.05. Abbreviations: CON, control group; CP, 5% citrus pomace group.

**Figure 6:**
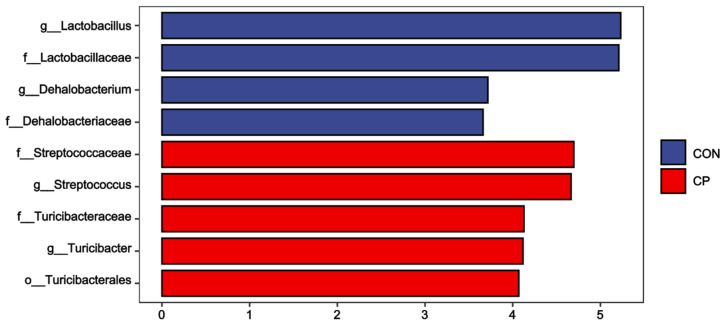
LEfSe analysis of colonic microbiota in Tibetan pigs (LDA scores > 3.0; n = 4). Abbreviations: CON, control group; CP, 5% citrus pomace group.

The colonic microbiota composition of Tibetan pigs fed the two experimental diets is presented in Figure 5. Compared to the control group, dietary inclusion of 5% citrus pomace reduced the abundances of *Lactobacillaceae* and *Lactobacillus*, while increasing the abundances of *Streptococcaceae*, *Turicibacteraceae*, *Streptococcus*, and *Turicibacter* in the colonic digesta of Tibetan pigs (*p* < 0.05). The LEfSe analysis was further conducted to assess alterations in specific bacterial taxa across taxonomic levels, including phylum (p), class (c), order (o), family (f), genus (g), and species (s), within the colonic digesta of Tibetan pigs fed the 5% citrus pomace-included diet vs. the control diet (Figure 6). The results show that, compared to the control group, Tibetan pigs fed diets containing 5% citrus pomace exhibited reduced abundances of *Lactobacillus* (g), *Lactobacillaceae* (f), *Dehalobacterium* (g), and *Dehalobacteriaceae* (f), while showing increased abundances of *Streptococcaceae* (f), *Streptococcus* (g), *Turicibacteraceae* (f), *Turicibacter* (g), and Turicibacterales (o) in the colonic digesta (*p* < 0.05).

## 4. Discussion

The primary objective of this study was to assess the impact of long-term dietary citrus pomace inclusion on growth performance, intestinal morphology, digestive enzyme activity, antioxidant status, and colonic microbiota in Tibetan pigs, thereby addressing the existing gap in research concerning the utilization of food by-products as feed ingredients. In this study, dietary inclusion of citrus pomace at 5%, 10%, and 15% did not adversely affect the ADG, ADFI, or FCR of Tibetan pigs during the 90-day feeding period. Importantly, dietary 5% citrus pomace inclusion decreased FCR of Tibetan pigs. The results align with previous research on weanling pigs, which demonstrated that supplementation with 7.5% citrus pulp did not compromise growth performance [17]. Additionally, studies on finishing pigs demonstrated no adverse effects from a 15% dried citrus pulp supplementation, while research on growing pigs indicated similar outcomes with a 10% inclusion rate [14]. Interestingly, previous studies also found that dietary inclusion of 2.5% and 5% citrus pomace did not adversely affect growth performance in growing pigs. However, increasing the inclusion rate to 10% resulted in compromised growth performance [18]. This phenomenon can be explained by the fact that higher concentrations of citrus pomace may increase dietary soluble non-starch polysaccharide levels, potentially impairing growth performance [19]. Therefore, based on growth performance, pigs from the control group and the 5% citrus pomace group were chosen for further evaluation of intestinal morphology, digestive enzyme activity in the small intestine, serum antioxidant capacity, and colonic microbiota.

The intestine is the primary site for nutrient digestion and absorption in pigs. A reduction in villus height accompanied by an increase in crypt depth often indicates intestinal stress, reflecting a suboptimal intestinal environment. In the current study, compared to the control group, dietary inclusion of 5% citrus pomace increased villus height in the duodenum and jejunum of Tibetan pigs. Similarly, morphological assessments revealed that citrus pulp supplementation increased villus height in the duodenum and jejunum in weaned piglets, as reported by Uerlings et al. (2021) [20]. Consistently, Zeng et al. (2022) observed increased ileal villus height and villus height-to-crypt depth ratio in weaned piglets fed diets supplemented with pomelo peel powder [21]. The improved intestinal morphology, which suggests an increased absorptive surface, further supports the lower FCR result in our study.

To further understand the changes in the digestive capacity of Tibetan pigs, we analyzed the activities of digestive enzymes, including α-amylase, β-amylase, sucrase, lipase, and neutral protease. The results showed that, compared to the control group, dietary inclusion of 5% citrus pomace significantly increased β-amylase activity in the duodenum and sucrase activity in the jejunum of Tibetan pigs. Additionally, lipase activity was elevated in the duodenum, jejunum, and ileum of Tibetan pigs fed the 5% citrus pomace diet relative to the control group.

The primary role of exocrine pancreatic secretion is to provide the enzymes necessary for the luminal digestion of carbohydrates, fats, and proteins. The pig’s pancreas secretes significant classes of digestive enzymes, including proteases and lipases, whose activities are crucial markers for assessing digestive capacity [22]. Furthermore, the intensity of sucrase activity, a pancreatic enzyme, serves as an indicator for evaluating intestinal development [23,24,25,26]. Overall, these results suggest that dietary inclusion of 5% citrus pomace positively influences the digestive capacity of the intestine by increasing the activities of key digestive enzymes such as β-amylase, sucrase, and lipase.

Citrus is abundant in bioactive compounds, including carotenoids, flavonoids, terpenes, and limonoids, which exhibit antioxidant properties [27]. In our study, compared to the control group, dietary inclusion of 5% citrus pomace significantly increased CAT activity in the serum of Tibetan pigs. Consistent with our findings, Zeng et al. (2022) demonstrated that dietary supplementation with 8 g/kg pomelo peel powder significantly increased serum CAT activity (11.00 vs. 7.25 U/mL) in weaned piglets compared to the control group [21]. Liu et al. (2024) also reported that dietary supplementation with 10% fermented or unfermented citrus pomace increased CAT activity in both serum and breast muscle of yellow-feathered broilers [28]. The findings further support our results that citrus pomace inclusion enhanced serum antioxidant status in Tibetan pigs.

The gut microbiome plays a pivotal role in maintaining gastrointestinal homeostasis, with diet being a significant factor influencing the gut microbiota [29]. In this study, a significant difference was found in β-diversity between treatment groups. Compared to the control group, dietary inclusion of 5% citrus pomace reduced the abundances of *Lactobacillaceae* and *Lactobacillus*, while increasing the abundances of *Streptococcaceae*, *Streptococcus*, *Turicibacteraceae*, and *Turicibacter* in the colonic digesta of Tibetan pigs. The LEfSe analysis further confirmed these findings. The enrichment of *Streptococcaceae* may be linked to the high fructose content in the citrus pomace-supplemented diet, a finding consistent with observations reported by Jones et al. (2019) [30]. *Streptococcaceae* and *Streptococcus* are documented to be associated with metabolic processes and gastrointestinal inflammatory responses [31,32]. It is proposed that the abundances of *Turicibacteraceae* and *Turicibacter* correlated with the production of short-chain fatty acids [33]. *Lactobacillaceae* and *Lactobacillus* are commonly recognized as probiotics [34,35], though some studies have documented their potential as pathogenic microorganisms [36]. Our findings indicate that long-term dietary supplementation may adversely impact the gut microbiota of pigs, as evidenced by reduced abundances of *Lactobacillaceae* and *Lactobacillus*. Therefore, the colonic microbiota of pigs responds to dietary citrus pomace inclusion in a complex manner.

This highlights that in vitro fermentation could be a viable strategy to address this issue [28,37]. Future research should focus on in vitro fermentation to provide fermented citrus pomace for pig nutrition.

Lastly, regarding the cost-effectiveness of citrus pomace in pig production, as an agricultural byproduct of citrus processing, moist citrus pulp (primarily from Navel oranges) has a relatively low market price. Although drying and storage incur additional costs, citrus pomace still offers a significant cost advantage compared to traditional feed ingredients. Thus, utilizing citrus pomace as swine feed presents considerable potential.

## 5. Conclusions

Dietary supplementation with 5% citrus pomace decreased feed conversion ratio, improved intestinal morphology, elevated digestive enzyme activity in the small intestine and serum antioxidant status, while having complex effects on colonic microbiota in Tibetan pigs. Our findings also highlight the potential application of citrus pomace in other commercial pig breeds. Future research should focus on in vitro fermentation to provide fermented citrus pomace as a feed ingredient for pigs.

## Figures and Tables

**Table 1:** Ingredient composition and nutrient level of the experimental diets (as-fed basis).

Item	The Inclusion Levels of Citrus Pomace			
	0	5%	10%	15%
Ingredient (%)				
Corn	56.8	61.5	57.0	52.5
Wheat bran	12.0	2.0	1.0	0.0
Rice bran	10.0	10.0	10.0	10.0
Soybean meal	10.0	13.5	14.0	14.5
Extruded soybean	6.2	4.0	4.0	4.0
Soybean oil	1.0	0.0	0.0	0.0
Citrus pomace ^1^	0.0	5.0	10.0	15.0
Premix ^2^	4.0	4.0	4.0	4.0
Total	100.0	100.0	100.0	100.0
Nutrient level ^3^				
Digestible energy, MJ/kg	12.98	12.94	12.94	12.90
Crude protein, %	15.00	14.93	14.95	14.97
Calcium, %	0.49	0.51	0.53	0.54
Total phosphorus, %	0.69	0.62	0.60	0.59
Available phosphorus, %	0.31	0.27	0.26	0.25
Lysine, %	0.68	0.70	0.70	0.70
Methionine, %	0.33	0.33	0.33	0.33
Threonine, %	0.33	0.33	0.33	0.33
Tryptophan, %	0.10	0.12	0.12	0.12

^1^ The nutritional composition of citrus pomace is as follows: dry matter, 87.10%; crude protein, 5.60%; ether extract, 2.10%; acid detergent fiber, 15.80%; calcium, 0.39%; phosphorus, 0.08%. ^2^ The premix offers the subsequent values per kilogram of diet: VA, 2000 IU; VD_3_, 200 IU; VE, 20 IU; VK_3_, 0.5 mg; VB_1_, 1.8 mg; VB_2_, 3.4 mg; VB_6_, 1.2 mg; VB_12_, 0.07 mg; pantothenic acid, 10 mg; niacin, 15 mg; biotin, 0.15 mg; folic acid, 0.4 mg; Fe, 110 mg; Cu, 4 mg; Mn, 3.0 mg; Zn, 70 mg; I, 0.14 mg; Se, 0.3 mg; ^3^ Calculated values.

**Table 2:** Effects of dietary inclusion with citrus pomace on growth performance of Tibetan pigs (n=4, mean ± SEM).

Item	The Inclusion Levels of Citrus Pomace				*p*-Value
	0	5%	10%	15%	
Initial body weight, kg	16.7 ± 0.7	16.6 ± 0.7	16.5 ± 0.5	16.7 ± 0.8	0.990
Final body weight, kg	48.2 ± 3.2	49.9 ± 1.8	50.7 ± 0.9	47.8 ± 2.8	0.150
Average daily feed intake, g	1213.0 ± 83.9 ^a^	1146.7 ± 35.5 ^a^	1326.0 ± 53.4 ^b^	1196.3 ± 57.8 ^a^	0.020
Average daily gain, g	349.6 ± 33.7	370.7 ± 34.2	380.2 ± 13.6	345.2 ± 24.9	0.120
Feed conversion ratio	3.48 ± 0.16 ^a^	3.11 ± 0.30 ^b^	3.49 ± 0.24 ^a^	3.40± 0.27 ^a^	0.047

^a,b^ Different letters indicate statistical differences among treatment groups (*p* < 0.05).

## Data Availability

The raw sequencing data of colonic microbiota were deposited in the National Center for Biotechnology Information (NCBI) with the accession number PRJNA1300782.
